# NSGC 2025 incoming presidential address: The power of choices: Your butterfly effect

**DOI:** 10.1002/jgc4.70115

**Published:** 2025-09-26

**Authors:** Sara Pirzadeh‐Miller

**Affiliations:** ^1^ Cancer Genetics Program University of Texas Southwestern Medical Center Dallas Texas USA

**Keywords:** incoming presidential address, leadership, professional development

## Abstract

The 2025 incoming presidential address outlines reflections on a personal leadership journey that positions the incoming president for success in this particular era, and presents a vision for advancing the genetic counseling profession. The development of leadership values in the journey were grounded in resilience, collaboration, and the integration of diverse perspectives. Building on this foundation, the address highlights forthcoming milestones that will shape the field. The achievement of Medicare recognition for genetic counselors is described as a pivotal step toward expanding patient access, promoting equity in genomic healthcare, and ensuring the sustainability of practice. Beyond reimbursement, the importance of securing representation at decision‐making tables is emphasized, with the goal of positioning genetic counselors to influence healthcare policy, genomic professional strategy, and clinical innovation. The vision also includes efforts to elevate public awareness, advancing the visibility of genetic counselors to establish the profession as widely recognized and valued across healthcare and society. Modernization of clinical practice is discussed as an essential priority to adapt to rapidly evolving genomic technologies, emerging care models with increasing patient demand, and better integration and sustainability throughout the national healthcare system. The address also underscores the organizational responsibility to create and sustain an environment of inclusion and belonging, fostering professional growth and engagement for all members. Together, these themes articulate a comprehensive framework for leadership and organizational action, aimed at strengthening the profession's role in delivering equitable, innovative, and patient‐centered genomic care in any setting of practice.

I still shock people when I tell them that I have loved genetics since learning about genetic engineering in 5th grade in the early 1990s. My dad, born and raised in Tehran, Iran, was a civil engineer at that time. At dinner one night, we talked about career paths. He started listing types of engineers given his own vocation. He said genetic engineer, and I was immediately intrigued. I even said in my 5th grade graduation book, when asked what I would be doing in the year 2012, that I would be a genetic engineer. I also thought we would be living in space.

I found the term “genetic counselor” in 7th grade biology career research, sitting on the library floor reading a book that I found on the topic. I loved what I learned! The story of a person's life and the powerful impact a genetic counselor could have on it with their unique skillset. I just knew. And in 2012, I was 7 years into my genetic counseling career.

A central dogma of my love of this profession has been how much I enjoy working with people and learning about their journey, their life story. That translates to the books, movies, and music I enjoy. I have always been fascinated by people and how their seemingly small choices lead them down a road that might not have been had they not made that choice.

This is essentially the “butterfly effect.” Seemingly miniscule events cause much larger events or outcomes to occur. For example, a butterfly's beating wings in its migration, while seemingly small, cause shifts in the atmosphere that result eventually in a tornado across the world.

In my own life, I think about this—what if I did not have that conversation with my dad? What if I did not do that research project in middle school where I found “genetic counselor”? All of the seemingly inconsequential tasks or conversations, in my mind, are essentially the butterfly effect in my own life and my subsequent road I followed.

The small choices people make that cause rippling effects in their journey are why I have always enjoyed the presidential address every year since I started attending NSGC as a second‐year student in 2004. I have loved to learn how a successful leader in our profession made their choices that got them to where they are. Inspiration and motivation to keep making smart choices on my own journey. I especially appreciated experiences shared about introducing their work to a prospective student that influenced one individual to consider genetic counseling. This one individual, in the future, will impact patients and future prospective students. The butterfly effect.

By the way, it is not lost on me that this is my 20th anniversary of being an NSGC member, and I never would have imagined all of my choices would have led me here in front of esteemed colleagues in the profession I love. As I remember myself sitting as a 2nd year student listening and thinking “that could never be me,” well, here I am, in front of you, telling you about my choices that created my journey. Just as I enjoyed previous presidential addresses to understand who our leader is and build trust in their leadership, it is important that all of you can get to know who I am for the same reason.

My aspiration to be a genetic counselor was still my focus as I went from that 7th grader sitting on the library floor through high school graduation. With this goal, I went to Texas A&M because it was one of the only universities in the nation with a true Genetics undergraduate degree. At that time, NO ONE there knew what a genetic counselor was, even in a Genetics department, and I had mentors guiding me towards MD/PhD routes in related fields. I made what turned out to be a very pivotal choice to apply for a competitive internship spot which allowed me to go to Washington DC for a science and public policy summer experience. I was a summer intern at the Association for Women in Science (AWIS). In this experience, I was able to attend Congressional meetings on interesting topics in science and experience life in the Beltway. It gave me a breather from my college environment and let me think again about genetic counseling and what I loved in the first place. It was that summer that I knew I was fully committed to pursuing this career and there was no turning back.

Fast forward, my move to UT Southwestern in Dallas/Fort Worth, Texas, to join the Simmons Cancer Center genetics program would be the choice that would have a true butterfly effect. I was lucky enough to get on the ground floor of an amazing cancer genetics program 17 years ago. I was initially hired by an amazing genetic counselor leader and my mentor of a lifetime, Linda Robinson, with a goal to grow our regional site in Fort Worth. This is where I learned about program development and leadership. Outreach and education were a huge part of my time in the beginning to grow our clinic base.

From that time, I was challenged to continue thinking outside the box, especially when we wanted to serve our local safety net hospital systems and needed to figure out how we would get the funding to do that. We made a choice to apply for a small grant to bring dollars for genetic testing to start our safety net hospital clinics, which led to pursuing a state clinical services grant. Several successful awards later, our program has done foundational work in North Texas and throughout the state to create better cancer genetic counseling and services access for our underserved populations. One example is the implementation of high‐risk patient identification processes that, in turn, will impact families for generations. The butterfly effect.

The success I spoke of just now is the downstream impact of “pilot projects,” which I enjoy very much. This also means I am comfortable with change and lean into it.

I believe you do not just rest on your laurels for what has worked but consider how you make choices to push forward. And maybe, just maybe, see gains that you would not have achieved otherwise. And if your pilot project fails and you encounter what feels like a door closing in your face, which has happened to me over the years, be open to the next best choice you can make and find another open door. Frame failure as an opportunity for the next success story.

Choices we have made over the years have created countless effects. There are several examples, but here are a few. One was initiating credentialing for GCs and billing for our services to create sustainability of our clinical program. Another was deciding to write a JEMF application during the pandemic. A particularly exciting example was the fight to start a genetic counseling training program at UT Southwestern, which this year I am so proud to say admits its inaugural class.

The choice I made to return to Dallas 17 years ago led to a great job opportunity which led to an amazing ongoing professional journey. Returning to Dallas also led to attending a Texas A&M football watch party—where the only open seat at the party was next to a handsome man. That choice to take that seat led to the love of a lifetime with my husband, Roy, and the creation of our beautiful family: our sons Maddux, Kellan, and Vaughan.

The choices in my professional life with my work family, and in my personal life with the family my husband and I built, have allowed the opportunity for me to stand in front of you today.

I like the fact that, as I embark on my 20th year as a genetic counselor in 2025, I still am on a road to which I cannot see the destination. Yet, regardless of what I can or cannot see, I have confidence that I am a leader that is well‐positioned to guide NSGC today. A leader with determination to make NSGC better; a leader with genuine passion for this profession and for all of us to have opportunities that make us better together; a leader with tenacity to put in the work. I hope to give you all the confidence in me today that I will apply this same persistence, this same tireless work ethic, all with a cool head under pressure, to my commitment to the vision and mission of NSGC.

Throughout the history of NSGC, many challenges have seemed so big, so difficult, that progress seemed improbable at times. As discussed in the fantastic presentation at the 2020 Annual Conference, Past President Wendy Uhlmann spoke about the NSGC's historical challenges (Uhlmann et al., 2020). An example included the passage of the Genetic Information Non‐Discrimination Act or GINA. Fast forward to now, a time when many of you have not practiced in an era where GINA was not the law of the land. However, without a doubt, we are in a current era of encountering significant challenges for our profession and organization. I have seen through the most recent membership feedback surveys the challenges that impact all of us. Standing here today, I want you to know that I stand with all of you. These challenges are exactly what I intend to be working with our Board towards resolution. These aspirational goals will no longer be challenges for the future, but rather, our normal.

I will speak about my vision on what I think are 3 critical goals for our organization: first, an NSGC defined by its culture of inclusivity and belonging; second, the term “genetic counselor” as a household name with a highly recognized and valued skillset; third, and perhaps the most critical issue, securing the sustainability of our profession through achieving Medicare recognition of genetic counselors. If you attended the State of the Society yesterday, our President Dr. Colleen Campbell spoke to our upcoming Strategic Plan, where you heard similar themes. I want to speak now more about where I see us going and where our choices must, and will, lead to.

Vision 1: NSGC is a healthy organization where inclusivity is the norm and anyone can belong. I mentioned towards the beginning of this address that my father is Iranian. What I did not add is that my mother is Norwegian, a blond and green‐eyed woman from Minnesota who grew up not having left the state until she was in high school. My mom, who met and married my dad in college 50 years ago this year, lived in Iran with my dad's family for 3 years while he served in the Iranian army. She learned Persian, also known as Farsi, as well as learned about the culture and Iranian cuisine. Even when they returned to the US during the Iranian Revolution, I grew up surrounded by the language and culture even though I never lived in Iran myself. However, outside of my family, I always felt not “Iranian enough” or “American enough,” essentially a less visible minority. My blended background felt like a major challenge when I was younger.

I now know my blended background is my superpower. I have lived experience that informs my passion for belonging and inclusivity. Within NSGC, there are 2% of members in the last Professional Status Survey that report Middle Eastern descent (NSGC, n.d.‐a) (Figure 1). On the surface, many might not guess my identity or background accurately to know that I reside in that 2%. While I do not claim to understand all experiences of those who identify similarly, my lived experience makes me feel to my core the importance of anyone, no matter what their identity or background, feeling safe in the NSGC community. We must continue our progress towards an NSGC that has truly realized an inclusive culture that weaves diversity, equity, inclusion, and belonging principles naturally into all that is done.

**FIGURE 1:**
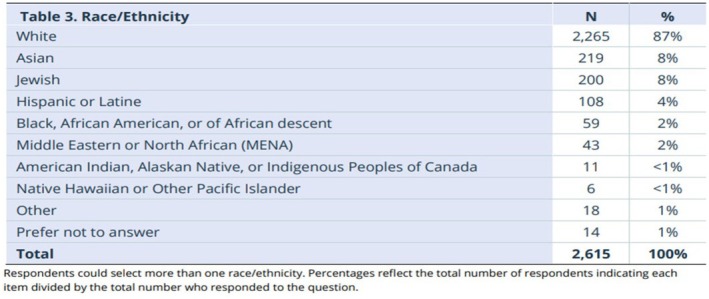
2024 PSS executive summary.

In the recent Exeter assessment, the feedback from members shows that while NSGC has done a lot of work to advance J.E.D.I. initiatives in the last 4 years, there is still much work to be done (NSGC, n.d.‐b) (Figure 2). The membership survey feedback says the same—continuing issues with members that do not feel safe and instead feel threatened within NSGC. To this point, in the 2024 membership survey, a quote in the comments resonated strongly with me: “I wish there was a safe, healthy way to all co‐exist as genetic counselors” (NSGC Membership Survey 2024). Sit with that for a second.

**FIGURE 2:**
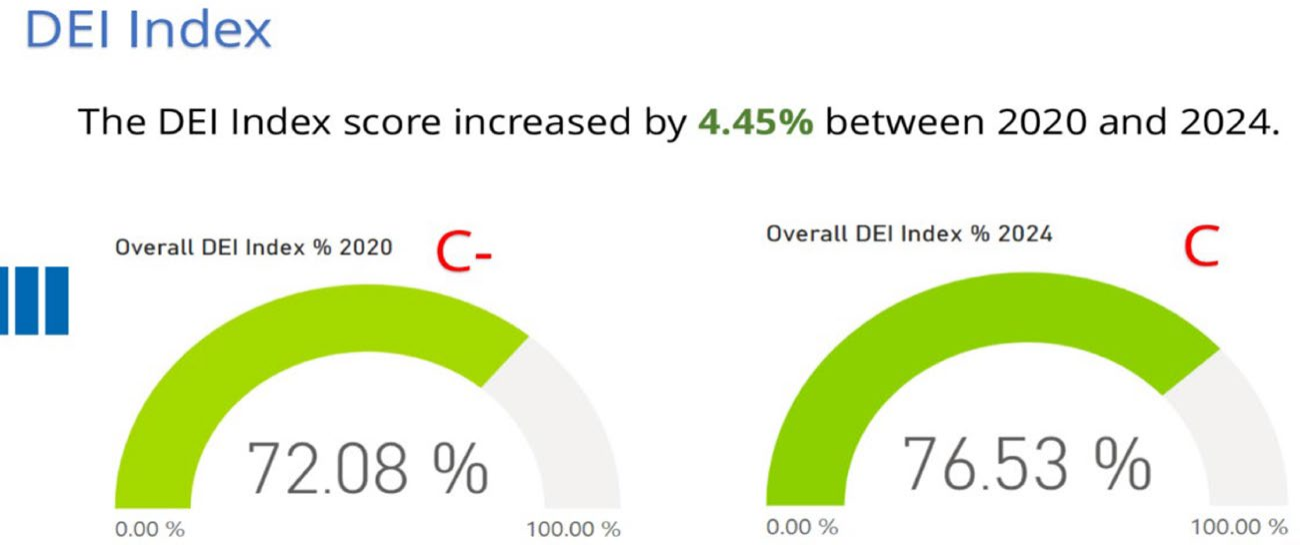
NSGC JEDI action plan—Exeter report.

NSGC continues to receive feedback from members of our community that are hurting, and that hurt impacts all of us. I have seen, as I know many of us have, discourse on our discussion forums that has a tenor of assumption of mal intent. Let me be clear—we all need to remember the mission that we share as genetic counselors when posting messages and digesting information. You would not be here if you were not all in on your purpose, your mission. I urge everyone before hitting “send” to think for a moment of words that cannot be taken back since an impression never leaves the minds of the reader. We all need to take personal responsibility and ownership for our communications and taking care of each other.

As my sons' elementary school motto says: “I take care of myself, I take care of others, and I take care of this place.” I look at that message and think of the beautiful simplicity of it. I see it as “others” are all of you as my colleagues, and “this place” is NSGC. If our elementary school children can abide, we as professionals and grown adults can too. I also think of this topic of conversation I have with my kids at home: how do we learn from everyone in various ways and take some responsibility in elevating each other's voices? I challenge everyone here in New Orleans and participating virtually today to find a colleague that has a position or experience wildly different from yours in any way. Learn one new perspective today. What effect could you create right now? Maybe that brief conversation and understanding leads to more conversations with others that inspires change.

Here at this conference this week, many of you have that opportunity very easily! Please use it and continue to challenge your networks to do the same. Post it on social media and imagine the impact you could have in sharing your learnings! I am SO excited to read it—feel free to tag me in it or use the NSGC conference hashtag! We will make a butterfly effect happen!

Vision 2: The genetic counselor skillset is valued, and the profession is a household name. Here's a question: Why are genetic counselors not asked for the first quote in genetics‐related stories in the media? Every time I see a national news outlet with a genetics story, it is usually other health care providers speaking to the genetic elements of the story. It is a pet peeve, honestly. Why aren't we there? The same argument applies to our patient populations—most do not think of us when genetic testing or evaluation comes in the care conversation. If they did, they would ask for us by name, even if their healthcare provider did not bring it up—“can I see a genetic counselor? What are my options?” We need to be a household name, so patients ask for us.

This also applies to genetic counselors in any type of role—industry, research, public health, to name a few. We must raise our visibility to show our skillset and how employers should be asking for someone who is trained as a genetic counselor because of the unique contributions we can make to their organizations. Feedback in the membership survey plainly states that members in the clinical and industry sectors are most concerned about the workforce, as well as public and name awareness of our profession. I believe these are related issues. The opportunities for those trained as genetic counselors are limitless, as our skills apply in infinite ways to any section of industry or healthcare. It is just up to us to make that evident, and we are our best advocates. We know what makes us as genetic counselors unique among other healthcare professionals, and this needs to be what we focus on in our evolving world.

I also think about how this skill set and visibility applies to research. While I am not someone who would identify myself as a “researcher,” I can appreciate the importance of how we as genetic counselors in our unique role improve outcomes for patients and populations we serve. We need to continue to produce the data to substantiate our impact and unequivocally show our value in genomic healthcare. However, a dearth of mechanisms for genetic counselor‐led research award opportunities exists.

Dr. Heather Zierhut and colleagues conclude in their recent publication on National Institutes of Health funded research awards, “Despite significant growth in genetic counseling research support from US‐based funders over the past 3 years, major gaps related to funding exist, including that most award recipients are not genetic counselors” (Zierhut et al., 2024).

To expand opportunities, NSGC does provide mechanisms, like the JEMF Award, which I have been honored to receive, and the NSGC Research Initiative, that I hope you budding researchers will take advantage of. I believe NSGC should help shepherd national conversations towards expanding research support for genetic counselor‐led work. To my colleagues with amazing research expertise, know how thankful I am to the many of you who have mentored people like me in contributing to the overall goal of more genetic counselor‐led research.

NSGC cannot raise the visibility of us alone. I want to challenge all of you to do all we can to make us a household name. Be a subject matter expert or volunteer to write a medical blog for your organization. Raise your hand with a great idea to boost genetic counselor expertise to your leadership. Each of us, with a small choice, can create cumulatively large effects that ripple across our profession and the world. The butterfly effect.

Vision 3: Genetic counselors are Medicare‐recognized providers, which solidifies sustainability. I am a genetic counselor leader who has spent my entire career so far in a clinic‐facing setting, and much of my career growing and advocating for who we are as genetic counselors and our value to patients, healthcare, and other settings. I will admit there was a time when I did not understand the importance of genetic counselor credentialing and billing. Our clinical practice did not bill and was not credentialed with insurers for many years. In 2019, I was tasked by my administration to initiate billing for our services. At the time, my team and I had a LOT of trepidation about the potential negative impacts of this on our community referral base, yet we made the choice to be drivers of positive change. With that, we have been able to show increasing revenue and positive return on investment for our institution that has allowed our program to continue growing even through a pandemic.

Through that experience, I can appreciate the importance of genetic counselors learning how to credential with their institutions and insurers, having the critical conversations with the right people in their billing infrastructure to navigate that road, and holding their institutions and themselves accountable to help with program growth when you show nothing is being left on the table. This work also translates to the sustainability of our entire profession with its diverse roles, not just the genetic counselors in direct patient care.

The more genetic counselors we can have in sustainable clinical programs, the more genetic testing laboratories and related industry settings are able to grow and flourish.

This contributes to the success of our GC colleagues that work in these settings. So the ability of GCs to bill appropriately for their services has ripple effects to GCs in many sectors and beyond this nation's borders.

Our membership feedback stated loudly and clearly that Medicare recognition is the top priority for NSGC, followed closely by workforce concerns and upcoming CPT code evolutions (NSGC Membership Survey, 2024). Through that feedback, I am glad to see that many of you recognize the importance of Medicare recognition. For those who are learning, let me give you a quick review. This recognition will allow payors who follow Medicare regulations to reimburse directly for genetic counselor services. It will change the entire game for our profession's sustainability, full stop. While I and others have heard members ask “why is the federal effort taking this long,” we have learned that we are on a very normal track compared to other professions who have dealt with the same issue. The take home message here: do not get complacent, be patient and persistent, and we must stay on this course with continued vigor.

I feel more confident now of our impending success in this endeavor with the data‐driven choice the Board made this year to start the process of identifying a new lobbying firm to refresh our federal strategy. But the federal efforts are not a silver bullet. At the same time, there is continued progress needed on the state licensure efforts, CPT code transition, and payor reimbursement. As we enter 2025, there will be much new terrain to navigate, and my top priority with our Board will be to get the federal bill and CPT transition across the finish line. Please be attentive to “calls to action” for our members as we continue this work. As the saying goes, teamwork makes the dream work!

Sustainability of our profession is multifaceted. We have discussed recognition and reimbursement, yet another piece of this is modernizing clinical service delivery.

I believe we need to preserve some historical tenets of genetic counseling, but at the same time, recognize where a cultural shift is necessary. We have lived in a place of “genetic exceptionalism” for a variety of reasons in our clinical practices. For example, many GCs engage frequently in conversations about test cost with patients. Do other healthcare providers have this same level of conversation about the cost of tests they order? No, they do not. I firmly believe we in the clinical‐facing space need to modernize our practice by aligning operations with other similar types of healthcare providers. This includes practicing top of scope. We are at a place in our profession's numbers and growth that this transition is possible in many settings. For example, genetic counselors at my institution were not a part of the Press‐Ganey patient satisfaction survey administered for most patient encounters. We worked to integrate ourselves in it, so we could have institutional metrics like other healthcare providers do, which also helps with our visibility in the healthcare system. As an added benefit, it shows how excellent our metrics are, which piques the positive interest of leadership!

Advanced practice providers like physician associates and nurse practitioners have successfully, in many cases, worked to optimize their clinical practices to operate independently where appropriate to increase patient access to visits. There are lessons to be learned by genetic counselors in this space. The sustainability of our profession is the gift that keeps on giving. Genetic counselors, in any setting, are impacting their patients, populations, and audiences of any kind that, in turn, affect their families and future generations around the world. The true definition of the butterfly effect.

When this vision is achieved, all of you and our future generations of genetic counselors will be the beneficiaries. NSGC is the destination for all genetic counselors to find their niche. The public knows who we are, what we do, and how unique we are. More opportunities for us in many industries because our skills are desired. Realizing a sustainable future.

There's nothing wrong with NSGC that cannot be fixed with what is right about NSGC.

I love challenges, and I know many of you do too. As I close this address, I will repeat the challenge I issued to you today: How will you use your choices, your power, to cause a butterfly effect that elevates your profession and its voices? I call on all of you today to think about how you can make that one small choice that will lead to change. Whether it is micro or macro impact, all of it matters. Maybe an opportunity is coming up related to a choice you make in the challenge today. Maybe this is the moment, THAT pivotal choice, that you look back on and think, “what if I had not done that?”

The beat of one butterfly's wings can create a tornado in a distant land. I hope each of you can be the beating wing that creates an avalanche of change for future generations of genetic counselors. Be the butterfly effect! Thank you.

## AUTHOR CONTRIBUTIONS

**Sara Pirzadeh‐Miller:** Visualization; writing – original draft; writing – review and editing.
